# Author Correction: Temporal structure of natural language processing in the human brain corresponds to layered hierarchy of large language models

**DOI:** 10.1038/s41467-026-72170-9

**Published:** 2026-04-17

**Authors:** Ariel Goldstein, Eric Ham, Mariano Schain, Samuel A. Nastase, Bobbi Aubrey, Zaid Zada, Avigail Grinstein-Dabush, Harshvardhan Gazula, Amir Feder, Werner Doyle, Sasha Devore, Patricia Dugan, Daniel Friedman, Michael Brenner, Avinatan Hassidim, Yossi Matias, Orrin Devinsky, Noam Siegelman, Adeen Flinker, Omer Levy, Roi Reichart, Uri Hasson

**Affiliations:** 1 Department of Cognitive and Brain Sciences, Hebrew University, Jerusalem, Israel; 2 Business School, Hebrew University, Jerusalem, Israel; 3 Google Research, Tel-Aviv, Israel; 4 Department of Psychology and the Neuroscience Institute, Princeton University, Princeton, NJ USA; 5 Bioinformatics Interdepartmental Program, University of California, Los Angeles, Los Angeles, CA USA; 6 New York University Grossman School of Medicine, New York, NY USA; 7 School of Engineering and Applied Science, Harvard University, Cambridge, MA USA; 8 Department of Psychology, Hebrew University, Jerusalem, Israel; 9 New York University Tandon School of Engineering, Brooklyn, NY USA; 10 Blavatnik School of Computer Science, Tel-Aviv University, Tel-Aviv, Israel; 11 Technion—Israel Institute of Technology, Haifa, Israel

Correction to: *Nature Communications* 10.1038/s41467-025-65518-0, published online 26 November 2025

In the version of this article initially published online, the colored plots in Fig. 3 were rendered incorrectly. The mistakes are in presentation and do not affect the results or conclusions of the study. For comparison, the original figure is available as Supplementary Information accompanying this amendment. The figure is now amended in the HTML and PDF versions of the article.

## Supplementary information

Original Fig. 3

