# Supplementary material for: Temporal structure of natural language processing in the human brain corresponds to layered hierarchy of large language models
Source: Nat Commun. 2025 Nov 26;16:10529. doi: 10.1038/s41467-025-65518-0 (PMC12657922; doi:10.1038/s41467-025-65518-0)
Supplement: Supplementary file 1 — Supplementary Information [file 41467_2025_65518_MOESM1_ESM.pdf]

Supplementary Tables

patient	**mSTG**	**aSTG**	**IFG**	**TP**
**1**	11**(negative)	6***	15***	0
**2**	0	0	2***	0
**3**	10*	6***	10***	4***
**4**	4***	0	1***	0
**5**	0	0	18***	1***(negative)
**6**	2	0	0	0
**7**	1**	1***	0	1**

Supp. Table 1: Distribution of electrodes per participant over the different ROIs. The significance addresses the correlation between the lag of the peak correlation and the index layer. Significance (one-sided): *$p < .05$ **$p < .01$ ***$p < .001$

**Phonological Category**	**Features**
Voicing	voiced =1, not voiced = 0
Manner of Articulation (Consonants)	nasal, plosive,non-sibilant-fricative,sibilant-affricate, sibilant-fricative,lateral-approximant,trill
Place of Articulation (Consonants)	bilabial,palato-alveolar,labio-velar,velar,approximant,labio -dental, palatal,dental,alveolar,glottal,labio-alveolar,uvular
Height (Vowels)	close, near-close,close-mid, mid,open-mid,near-open,open
Backness (Vowels)	front, near-front,central,near-back,back
Roundedness (Vowels)	rounded = 1, unrounded = 0

Supp. Table 2: Phonological Features

NO.	Neuropsych Score	Pathology/Epilepsy type/Seizure Focus	Implant
5	VCI: 145 POI: 96 PSI: 86 WMI: 95	Focal epilepsy arising from the left hemisphere with a broad focus involving the left temporal neocortex (superior, middle, inferior temporal gyri) left frontal operculum, inferior postcentral gyrus, insula	Left grid and strips
6	VCI: 87 POI: 123 PSI: 97 WMI: 89	Left temporal lobe epilepsy. Ictal onsets localized to the left temporal lobe (perilesionally) and left posterior mesial temporal lobe.	Left grid, strips, and depth electrodes
4	VCI: 100 POI: 109 PSI: 92 WMI: 86	Focal epilepsy localized to the left posterior insula and periopercular region at the frontoparietal junction.	Left grid, strips, and depth electrodes
3	not found	Probable focal epilepsy, not clearly lateralized or localized. ICEEG showed definitively that disabling clinical events were psychogenic non-epileptic attacks.	Left grid, strips, and depth electrodes
7	VCI: 96 POI: 79 PSI: 81 WMI: 86	Left hemispheric multilobar epilepsy	Left grid, strips, and depth electrodes
2	not found	Bilateral mesial temporal lobe epilepsy	Bilateral strips and depth electrodes
1	VCI: 107 POI: 104 PSI: 111 WMI: 114	Right anteromesial temporal lobe epeilpsy. ICEEG localized ictal onsets to the right temporal pole and right hippocampus	Bilateral strips and depths, and a left grid

Supp. Table 3: Additional information about patient pathology and neuropsychological scores.

LAYER INDEX	P-VALUE	Q-VALUE	LAYER INDEX	P-VALUE	Q-VALUE
1	0.784826	0.459996	25	0.731631	0.3963
2	0.061834	0.015458	26	0.719935	0.360514
3	0.016337	0.000953	27	0.608419	0.278859
4	0.016337	0.00111	28	0.569881	0.249323
5	0.409457	0.153546	29	0.38613	0.136755
6	0.016337	0.001688	30	1	0.949051
7	0.016337	0.001847	31	0.990003	0.696491
8	0.035199	0.0066	32	1	0.906098
9	0.016522	0.002409	33	1	0.94135
10	0.023182	0.003864	34	1	0.922248
11	0.016337	0.002042	35	1	0.9526
12	0.051168	0.01066	36	0.719935	0.374966
13	0.283744	0.094581	37	0.927577	0.618385
14	0.276009	0.086253	38	1	0.844096
15	0.21497	0.0627	39	0.784826	0.474165
16	0.059726	0.013687	40	0.927577	0.61807
17	0.016337	0.000372	41	0.820915	0.513072
18	0.191238	0.051794	42	1	0.961332
19	0.425111	0.168273	43	1	0.736217
20	0.990003	0.701252	44	1	0.942203
21	1	0.993479	45	0.548249	0.228437
22	0.749748	0.421733	46	1	0.968246
23	0.703456	0.337073	47	1	1
24	1	0.825196	48	1	0.907848

Supp. Table 4: The p-value and FDR-corrected q-value of the paired sampled t-test comparing the lags that achieve maximal correlation in the encoding across the different layers (n=48) of GPT2-XL.

	PREDICTED	NOT PREDICTED	ALL
mSTG	22	26	22
TP	22	26	22
aSTG	17	22	17
ifg	24	20	16

Supp. Table 5: Layers that maximize encoding performance for different combinations of ROI and word classification

Supplementary Figures

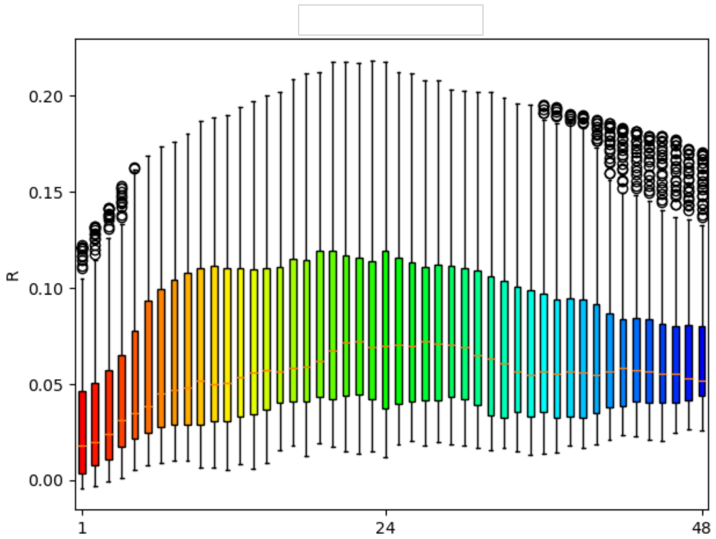

Supp. Figure 1: Here we plot Fig. 1B but as a boxplot to make the distribution across lags more clear. The bottom and top box edges correspond to the first and third quartiles (Q1 and Q3) respectively, and and the line in the middle is the median. The top whisker extends to the lag whose correlation is highest but still within Q3 plus 1.5 times the interquartile range IQR).The bottom whisker extends to the lag whose correlation is smallest but still within Q1 minus 1.5 times the IQR. The boxes are color coded by layer index (red-blue) as described in Fig. 1

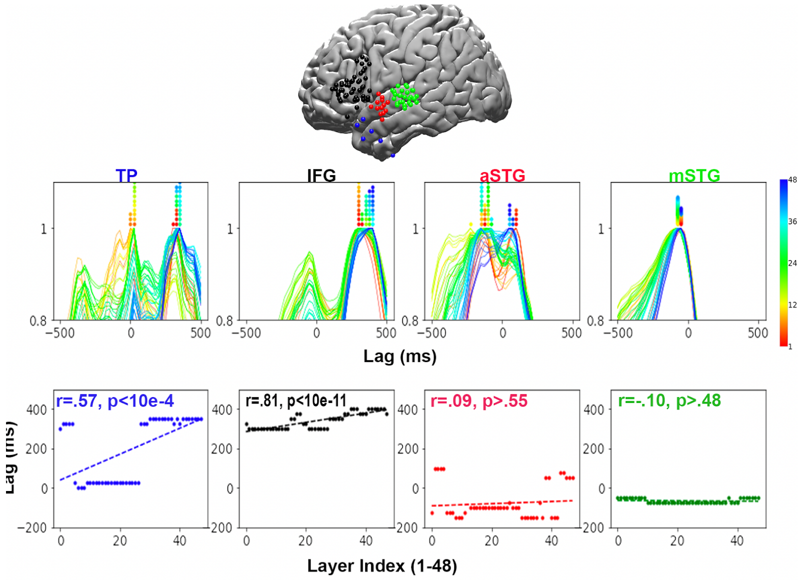

Supp. Figure 2: Temporal hierarchy along the ventral language stream for not predicted words. (Top) The location of electrodes on the brain is color-coded by roi, with blue, black, red, and green corresponding to TP, IFG, aSTG, and mSTG, respectively. (Middle) Scaled encoding performance for these ROIs. Color coded by layer index (red-blue) as described in Fig. 1. (Bottom) Scatter plot of the lag that yields peak encoding performance for each layer. One-sided p-values for the lag-layer correlations are reported (N=48).

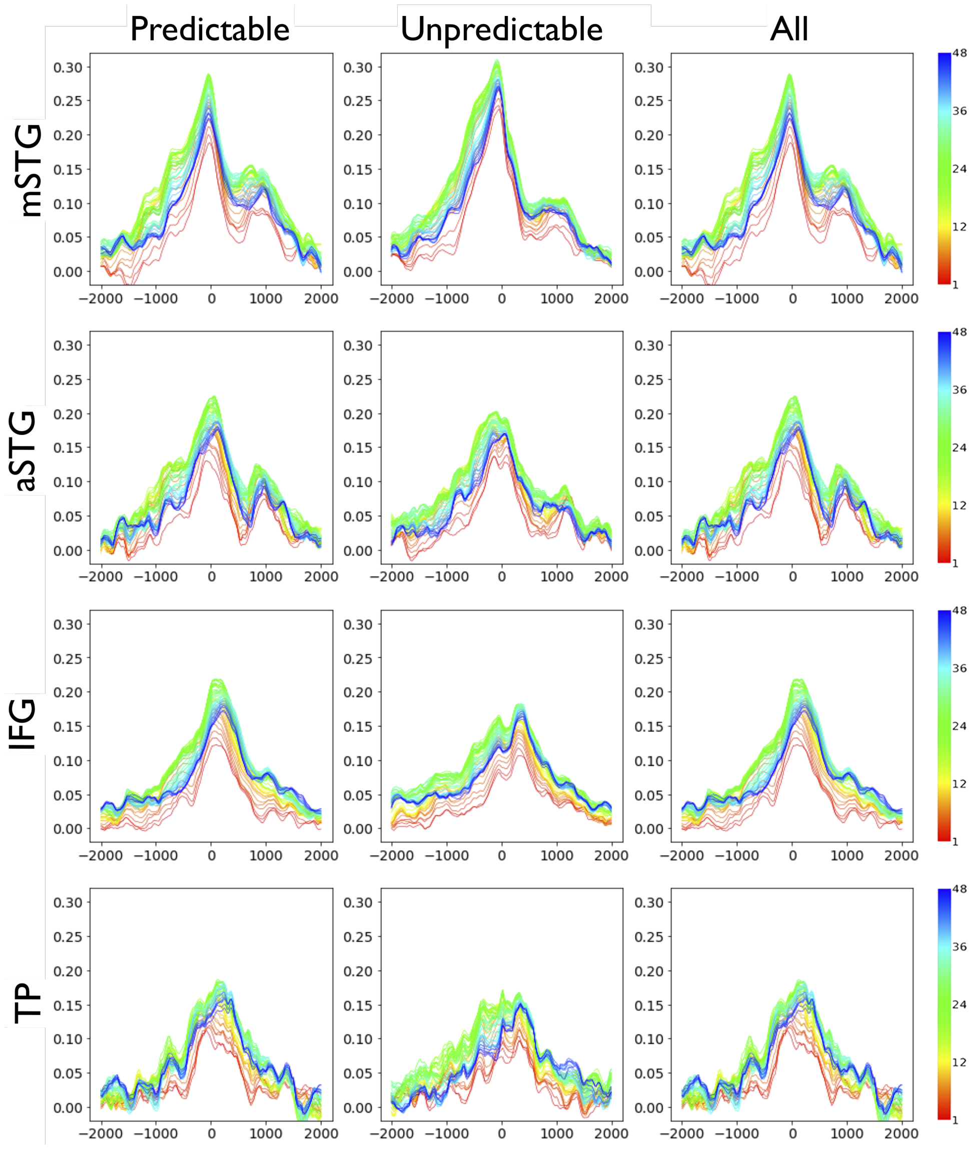

Supp. Figure 3: Encoding averaged over electrodes for each combination of layer (1-48), brain area (mSTG, aSTG, IFG, and TP), and word class ification (predicted, not predicted, all words).

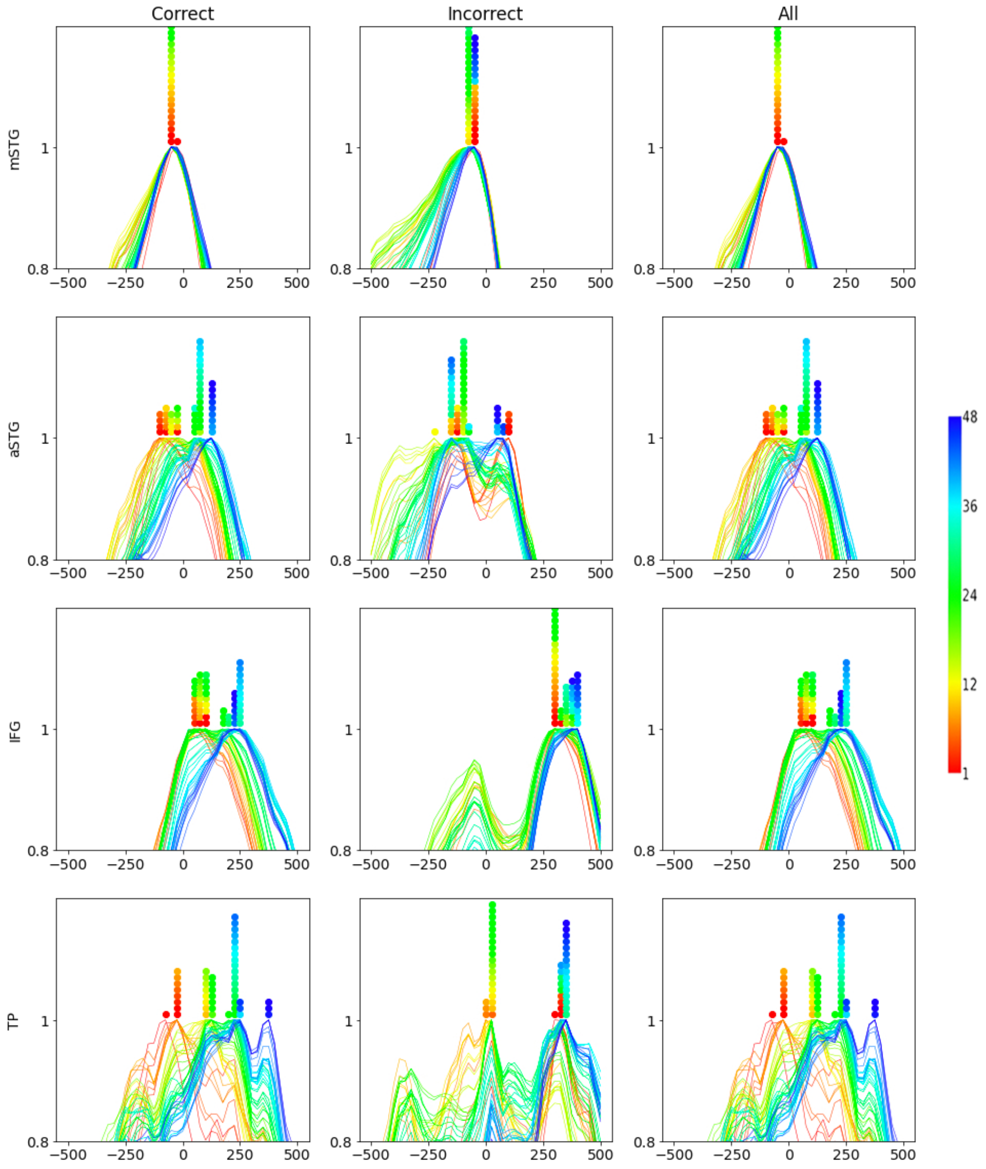

Supp. Figure 4: Scaled encoding for each combination of layer (1-48), brain area (mSTG, aSTG, IFG and TP) and word classification (predicted, not predicted, all words). For completion the correlation between the layer index and max-lag for condition 'All': mSTG (r=.56, p < 10e-4), aSTG (r=.81, p<10e-11), IFG (r=.89 ,p<10e-16), TP (r=.75 ,p<10e-9).

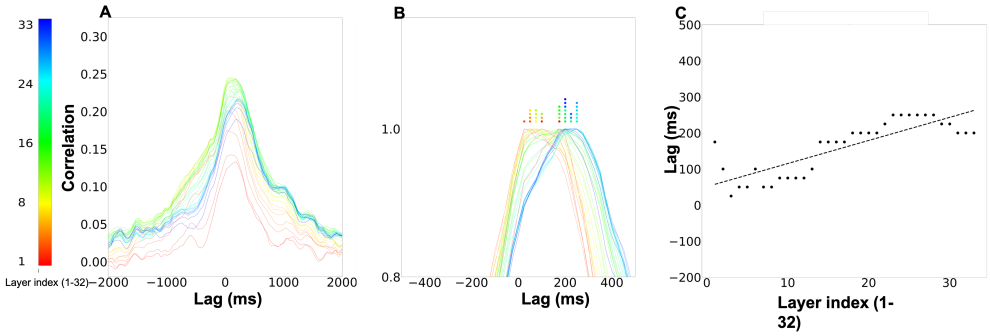

Supp. Figure 5: Replicating the hierarchy results using SOTA LLM, Llama-2 (A) Per-layer encoding plot in the IFG. (B) Scaled encodings. (C) Scatter plot of the lag that yields peak encoding performance for each layer.

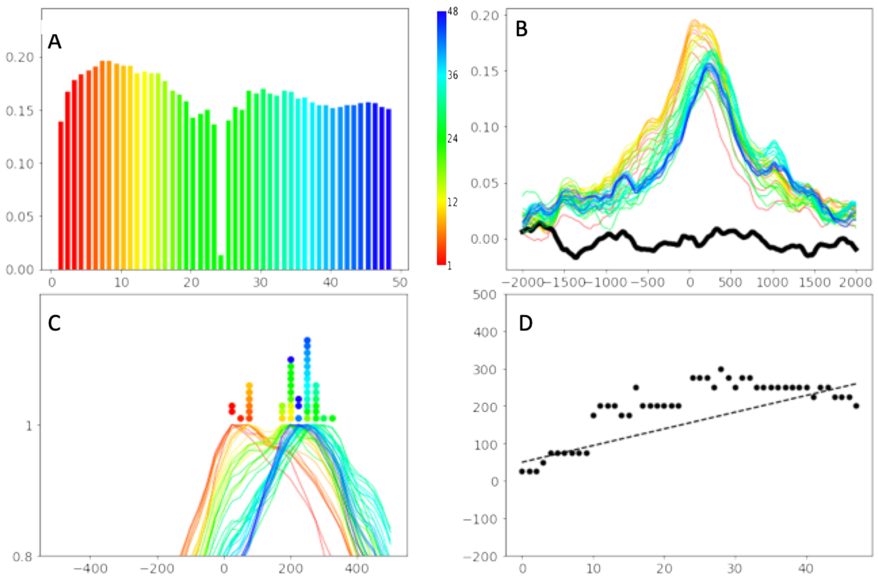

Supp. Figure 6: Replicating the findings regarding the IFG controlling for the correlation between the optimal layer (layer 22) and the other layers. We projected the best-performing embedding out of the embeddings at all other layers and then repeated our analyses. (A) Correlation between the brain and different embeddings is preserved after controlling for the variance explained in other layers by the optimal layer (layer 22).(B) The encoding for each layer after controlling for the variance explained by the optimal layer. The encoding by the optimal layer, shown in black, represents the residual signal after projecting out the variance that can be explained by the same layer (close to 0). (C–D). The temporal relation between the optimal encoding performance for lag and layer index is also preserved after controlling for the correlation between the optimal layer and other layers.

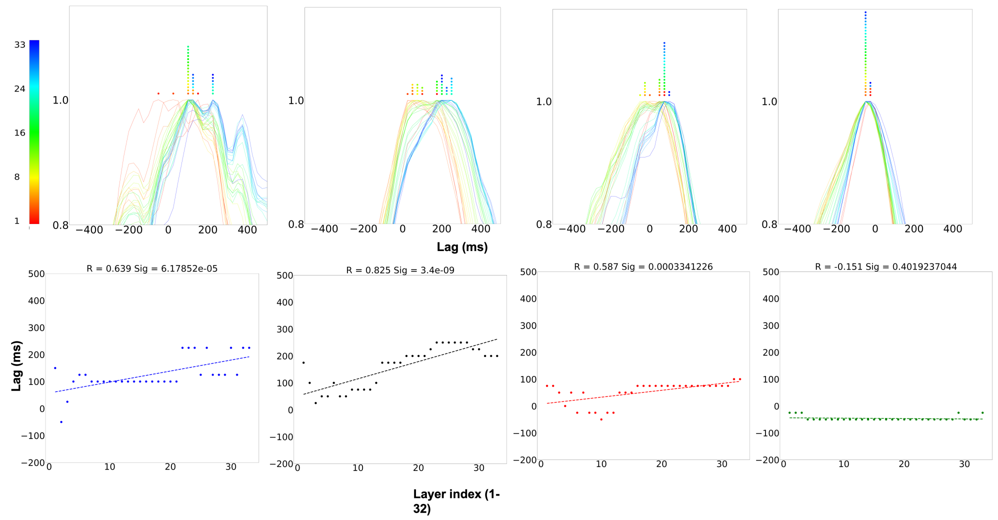

Supp. Figure 7: Replicating the hierarchy over the ventral stream using SOTA LLM, Llama-2. (Top) Scaled encoding performance for these ROIs. (Bottom) Scatter plot of the lag that yields peak encoding performance for each layer. Lag-layer correlations are reported along with associated p-values (N=48).

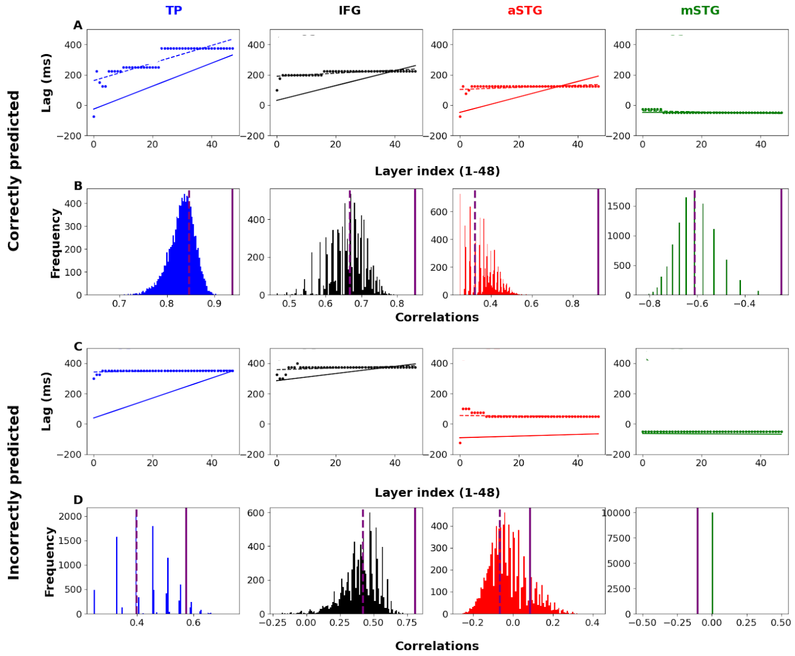

Supp. Figure 8: Control analysis for the alternative hypothesis that the lag-layer correlation we observe is due to a more rudimentary network property in which early layers represent the previous word, late layers represent the current word, and intermediate layers carry a linear interpolation between these words. (A) The lag-layer slope for the evenly spaced pseudo-layers (dashed line) is compared to the actual lag-layer analysis (solid line) for predicted words. The dots correspond to the lags of maximum correlation for the pseudo-layers. The dots for the actual layers are omitted for clarity. (B) Distribution of correlations induced by $10,000$ iterations of sampling pseudo interpolated layers. The vertical dashed purple line is the correlation achieved in the even-space case, and the continuous purple line represents the values achieved by the actual correlation. For completion, we present the same analyses and plots for not predicted words in C & D.

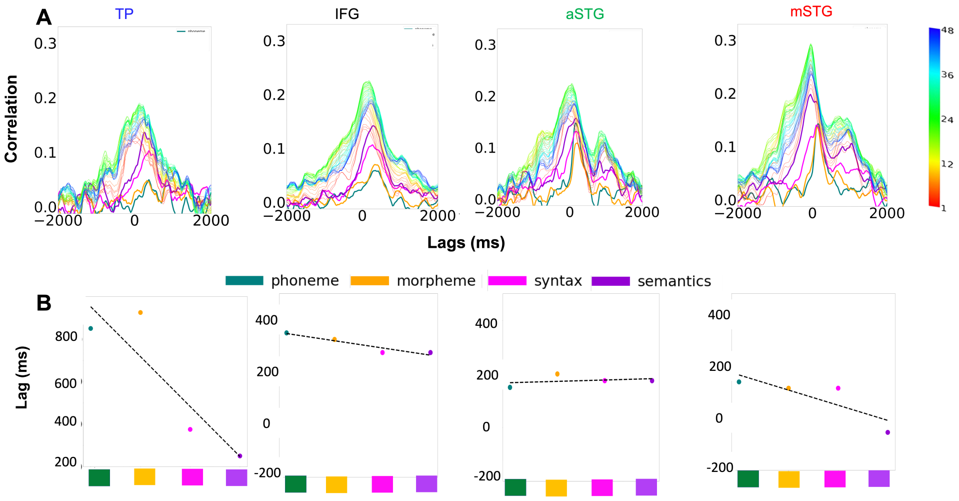

Supp. Figure 9: Layer-wise encoding models for all words. (A) Comparing the encoding analyses for different ROIs using embeddings induced by LLM (GPT2-XL; represented with thin lines) revealed higher correlations than those obtained with the curated embeddings induced by the four-level hierarchy of classical psycholinguistics (represented with thick lines). (B) The peak correlations of the embeddings, which were induced by the hierarchy used in classical psycholinguistics, did not appear to correlate with the temporal dynamics of the neural response.

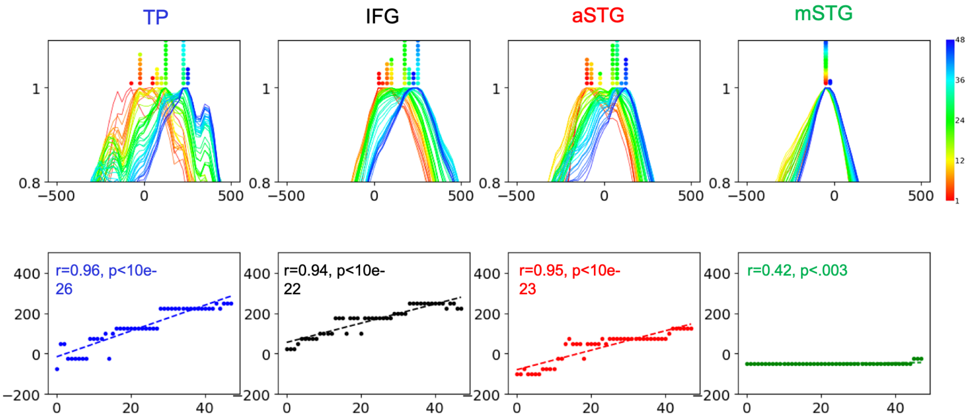

Supp. Figure 10: In our main results (Fig. 2 and Fig. 3), we trained PCA on the entire set of embeddings for a given layer and predictability condition (training and test folds). We used the resulting projection matrix to reduce the embedding size to 50. To verify our results still hold without leakage, we trained PCA on only the training folds and used the resulting projection matrix to reduce the embedding size to 50 (we repeated this process for each of the 10 training folds). On top, we show the scaled encodings for each ROI, and on bottom, we show the relationship between layer and lag of peak encoding correlation. For the bottom row, we report the lag-layer correlation and associated p-value (N=48).

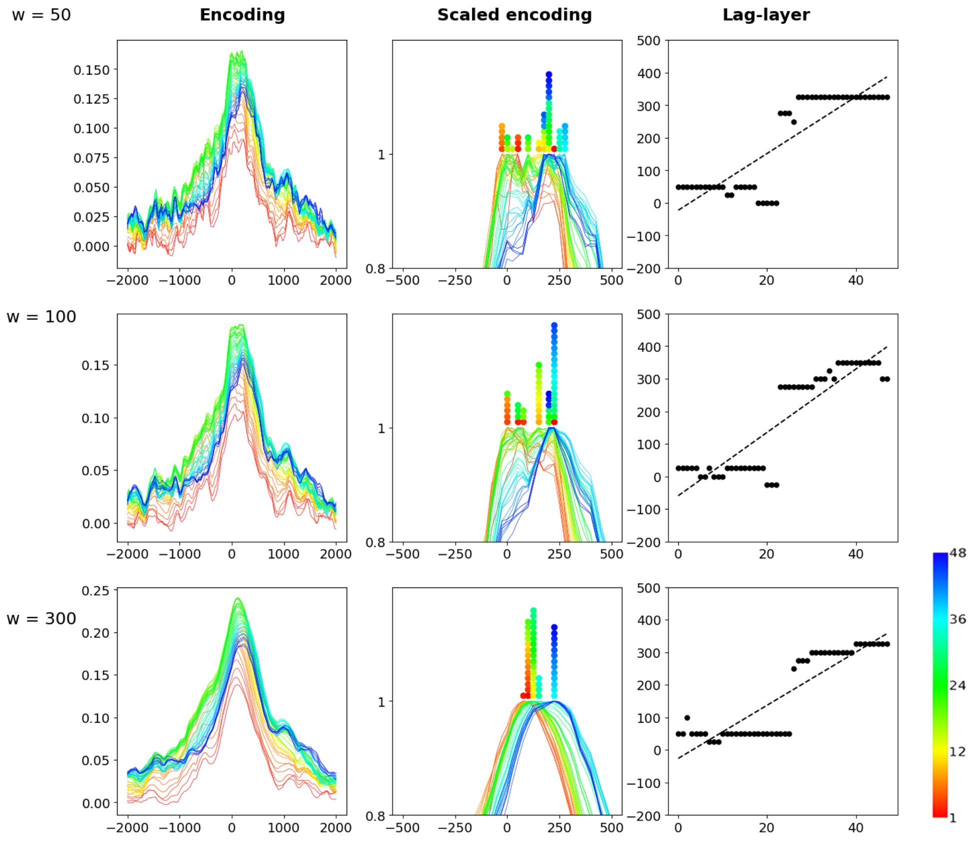

Supp. Figure 11: We re-ran the encoding analysis, scaled encoding analysis and lag-layer analysis for different smoothing window sizes (windows of 50, 100, 300). The lag-layer correlations are all positive (r>0.75) and significant (p<1e-10)

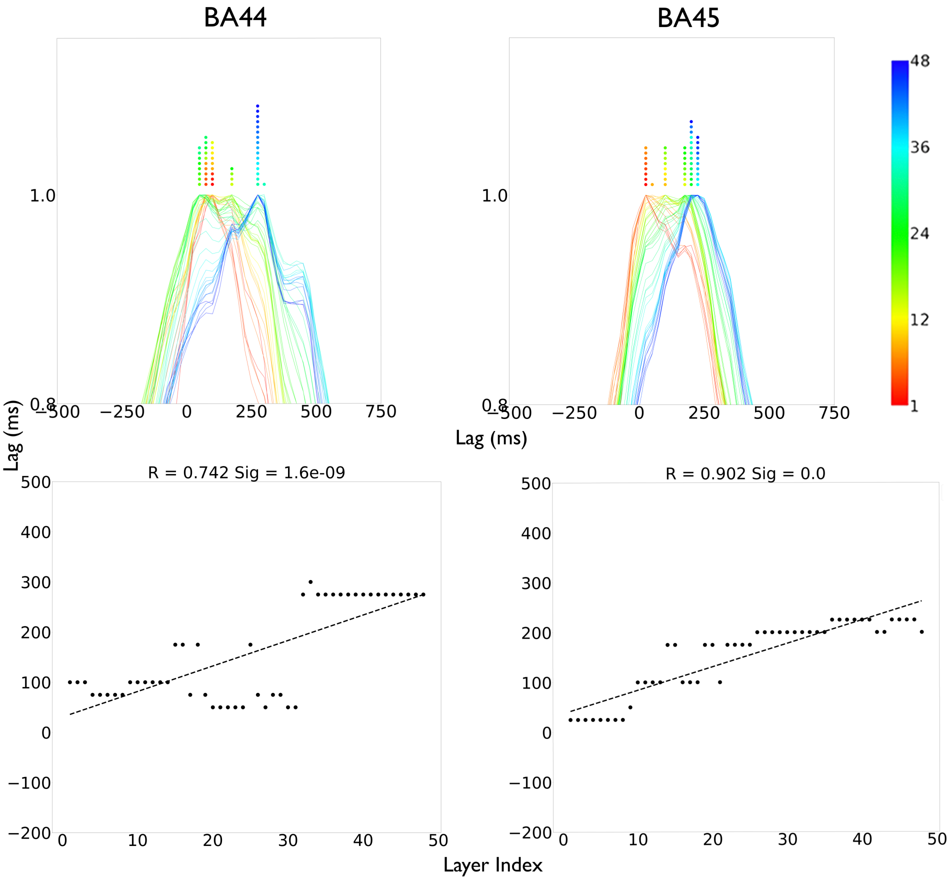

Supp. Figure 12: Timing of peak correlations across BA44 and BA45. Encoding (top) and peak lag values per layer (bottom) are shown for BA44 (left) and BA45 (right). We statistically compared the peak lags between the two IFG subregions (BA44: M = 155 ms, SD = 95; BA45: M = 152 ms, SD = 72). A paired-sample t-test revealed no significant difference (t(47) = 0.26, p = .79), and a Bayesian paired-sample t-test yielded a Bayes factor of 6, providing no strong evidence for a difference in timing between these subregions. In the bottom plots we report lag-layer correlations and associated p-values (N=48)
