# Supplementary material for: Temporal structure of natural language processing in the human brain corresponds to layered hierarchy of large language models
Source: Nat Commun. 2025 Nov 26;16:10529. doi: 10.1038/s41467-025-65518-0 (PMC12657922; doi:10.1038/s41467-025-65518-0)
Supplement: Supplementary file 2 — Reporting Summary [file 41467_2025_65518_MOESM2_ESM.pdf]

nature portfolio

Corresponding author(s): Ariel Goldstein

Last updated by author(s): Aug 26, 2025

Reporting Summary

Nature Portfolio wishes to improve the reproducibility of the work that we publish. This form provides structure for consistency and transparency in reporting. For further information on Nature Portfolio policies, see our Editorial Policies and the Editorial Policy Checklist.

Statistics

For all statistical analyses, confirm that the following items are present in the figure legend, table legend, main text, or Methods section.

n/a	Confirmed	
☐	☒	The exact sample size (*n*) for each experimental group/condition, given as a discrete number and unit of measurement
☐	☒	A statement on whether measurements were taken from distinct samples or whether the same sample was measured repeatedly
☐	☒	The statistical test(s) used AND whether they are one- or two-sided<br>*Only common tests should be described solely by name; describe more complex techniques in the Methods section.*
☒	☐	A description of all covariates tested
☒	☐	A description of any assumptions or corrections, such as tests of normality and adjustment for multiple comparisons
☐	☒	A full description of the statistical parameters including central tendency (e.g. means) or other basic estimates (e.g. regression coefficient) AND variation (e.g. standard deviation) or associated estimates of uncertainty (e.g. confidence intervals)
☐	☒	For null hypothesis testing, the test statistic (e.g. *F*, *t*, *r*) with confidence intervals, effect sizes, degrees of freedom and *P* value noted<br>*Give P values as exact values whenever suitable.*
☒	☐	For Bayesian analysis, information on the choice of priors and Markov chain Monte Carlo settings
☒	☐	For hierarchical and complex designs, identification of the appropriate level for tests and full reporting of outcomes
☐	☒	Estimates of effect sizes (e.g. Cohen's *d*, Pearson's *r*), indicating how they were calculated

Our web collection on statistics for biologists contains articles on many of the points above.

Software and code

Policy information about availability of computer code

Data collection	Recordings from grid, strip and depth electrode arrays were acquired using one of two amplifier types: NicoletOne C64 clinical amplifier (Natus Neurologics), band-pass filtered from 0.16–250 Hz, and digitized at 512 Hz; NeuroWorks Quantum Amplifier recorded at 2,048 Hz, high-pass filtered at 0.01 Hz and then resampled to 512 Hz.
Data analysis	Data were preprocessed using Matlab 2019b and The Fieldtrip toolbox (commit: 56769ab0s).<br>The Python packages used: Scipy v1.11.4, Statsmodels v0.14.1, nltk 3.9.1. Codes for:<br>For embedding extraction: https://github.com/hassonlab/247-pickling/tree/whisper-paper-1<br>For encoding models: https://github.com/hassonlab/247-encoding/tree/whisper-paper-1<br>For plotting: https://github.com/hassonlab/247-plotting/blob/main/scripts/tfspaper_whisper.ipyn

For manuscripts utilizing custom algorithms or software that are central to the research but not yet described in published literature, software must be made available to editors and reviewers. We strongly encourage code deposition in a community repository (e.g. GitHub). See the Nature Portfolio guidelines for submitting code & software for further information.

Data

Policy information about availability of data

All manuscripts must include a data availability statement. This statement should provide the following information, where applicable:

- Accession codes, unique identifiers, or web links for publicly available datasets
- A description of any restrictions on data availability
- For clinical datasets or third party data, please ensure that the statement adheres to our policy

The dataset is available in the following public GitHub repository: https://github.com/DVader96/GPTology

Research involving human participants, their data, or biological material

Policy information about studies with human participants or human data. See also policy information about sex, gender (identity/presentation), and sexual orientation and race, ethnicity and racism.

Reporting on sex and gender	Nine patients (5 female, 20–48 years old). Sex or gender are not relevant variables to this study, and analysis was not done considering it.
Reporting on race, ethnicity, or other socially relevant groupings	Nine patients (5 female, 20–48 years old). Sex or gender are not relevant variables to this study, and analysis was not done considering it.
Population characteristics	All the patients suffer from drug immune epilepsy. We select patients with intact cognitive faculty as determined by neuropsychological tests that are done as part of the hospitalization process. Effects were not moderated by age.
Recruitment	.Each participant provided informed consent following protocols approved by the New York University Grossman School of Medicine Institutional Review Board. Patients were informed that participation in the study was unrelated to their clinical care and that they could withdraw from the study without affecting their medical treatment. As the patients volunteer to the experiment there is a potential to self-selection based on this trait. However, the researcher could not think how it could impact the results.
Ethics oversight	The study was approved by the NYU Grossman School of Medicine Institutional Review Board (approved protocol s14-02101) which operates under NYU Langone Health Human Research Protections and Princeton University's Review Board (approval protocol 4962). Studies are performed in accordance with the Department of Health and Human Services policies and regulations at 45 CFR 46. Before obtaining consent, all participants were confirmed to have the cognitive capacity to provide informed consent by a clinical staff member. Participants provided oral and written informed consent before beginning study procedures. They were informed that participation was strictly voluntary, and would not impact their clinical care. Participants were informed that they were free to withdraw participation in the study at any time. All study procedures were conducted in accordance with the Declaration of Helsinki. Board/committee (Princeton university IRB) that approved the protocol: Daniel Notterman (Chair), Calvin Chin,Elizabeth Davis,Patricia Fernandez-Kelly,Michael Graziano, Kyle Jamieson, Jacqueline Kariithi, Vikki Lovvoll, Brandt McCabe, Chad Pettengill, Pascale Poussart, Naila Rahman, Rusty Reeves, Carrie Siegler, Jordan Taylor, Rory Truex

Note that full information on the approval of the study protocol must also be provided in the manuscript.

Field-specific reporting

Please select the one below that is the best fit for your research. If you are not sure, read the appropriate sections before making your selection.

☐ Life sciences ☒ Behavioural & social sciences ☐ Ecological, evolutionary & environmental sciences

For a reference copy of the document with all sections, see nature.com/documents/nr-reporting-summary-flat.pdf

Behavioural & social sciences study design

All studies must disclose on these points even when the disclosure is negative.

Study description	We used the recorded neural activity of four participants using dense intracranial arrays while they stayed in the hospital. This is a quantitative research as we correlate the brain signal recorded with the representation induced by deep ASR model (whisper)
Research sample	Nine patients (5 females, M = 31.43 years old, SD=10.13. Sex or gender are not relevant variables to this study, and analysis was not done considering it.

Sampling strategy	Each participant provided informed consent following protocols approved by the New York University Grossman School of Medicine Institutional Review Board. Patients were informed that participation in the study was unrelated to their clinical care and that they could withdraw from the study without affecting their medical treatment.<br>As the patients volunteer to the experiment there is a potential to self-selection based on this trait. However, the researcher could not think how it could impact the results.
Data collection	Recordings from grid, strip and depth electrode arrays were acquired using one of two amplifier types: NicoletOne C64 clinical amplifier (Natus Neurologics), band-pass filtered from 0.16–250 Hz, and digitized at 512 Hz; NeuroWorks Quantum Amplifier recorded at 2,048 Hz, high-pass filtered at 0.01 Hz and then resampled to 512 Hz.<br>The podcast was played using a laptop, by a research assistant that was blind to the experimental purpose. No additional personnel attended the experiment<br><br>The podcast was played using a laptop, by a research assistant that was blind to the experimental purpose. No additional personnel attended the experiment.<br><br>As the patients volunteer to the experiment there is a potential to self-selection based on this trait. However, the researcher could not think how it could impact the results.
Timing	Each patient came to the hospital at 2019. The recording was continous during their stay. The exact date of collection is an identifiable information and should not be shared: https://privacyruleandresearch.nih.gov/pr_08.asp
Data exclusions	No data was excluded
Non-participation	No patients were removed.
Randomization	As analyses can be seen for each participant (seperatly and together) there is no between particiapant analysis nor allocation to conditions.

Reporting for specific materials, systems and methods

We require information from authors about some types of materials, experimental systems and methods used in many studies. Here, indicate whether each material, system or method listed is relevant to your study. If you are not sure if a list item applies to your research, read the appropriate section before selecting a response.

Materials & experimental systems

n/a	Involved in the study
☒	☐ Antibodies
☒	☐ Eukaryotic cell lines
☒	☐ Palaeontology and archaeology
☒	☐ Animals and other organisms
☒	☐ Clinical data
☒	☐ Dual use research of concern
☒	☐ Plants

Methods

n/a	Involved in the study
☒	☐ ChIP-seq
☒	☐ Flow cytometry
☒	☐ MRI-based neuroimaging

Plants

Seed stocks	N/a
Novel plant genotypes	n/a
Authentication	n/a
