# Supplementary material for: Author Correction: Temporal structure of natural language processing in the human brain corresponds to layered hierarchy of large language models
Source: Nat Commun. 2026 Apr 17;17:3580. doi: 10.1038/s41467-026-72170-9 (PMC13090351; doi:10.1038/s41467-026-72170-9)
Supplement: Supplementary file 1 — Original Fig. 3 [file 41467_2026_72170_MOESM1_ESM.pdf]

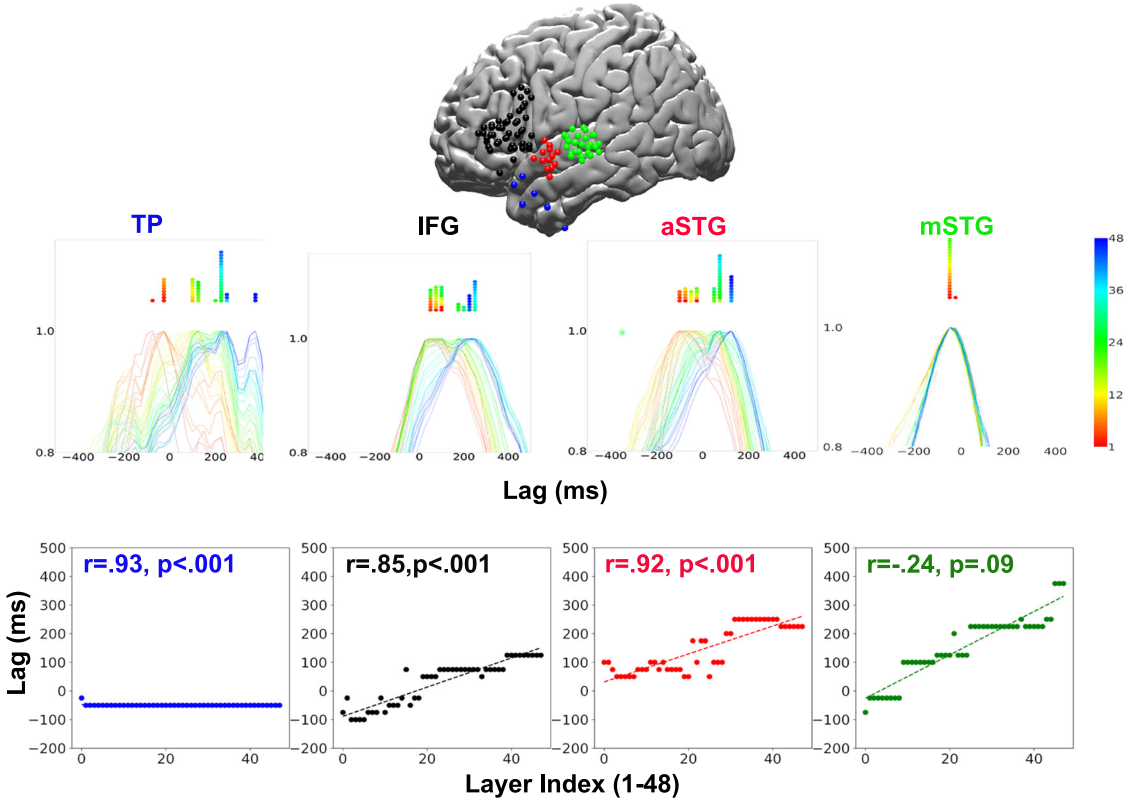

Fig. 3 | Temporal hierarchy along the ventral language stream for correctly predicted words. (Top) Location of electrodes on the brain, color-coded by ROI with blue, black, red, and green corresponding to TP, IFG, aSTG, and mSTG, respectively. (Middle) Scaled encoding performance for these ROIs. Color coded by layer index (red-blue) as described in Fig. 1. (Bottom) Scatter plot of the lag that yields peak encoding performance for each layer. One-sided p values for the lag-layer correlation are reported ($N = 48$).
